# Time-Dependent Damage Investigation of Rock Mass in an *In Situ* Experimental Tunnel

**DOI:** 10.3390/ma5081389

**Published:** 2012-08-16

**Authors:** Quan Jiang, Jie Cui, Jing Chen

**Affiliations:** State Key Laboratory of Geomechanics and Geotechnical Engineering, Institute of Rock and Soil Mechanics, Chinese Academy of Science, Wuhan 43007, China; E-Mails: cuijiewk@163.com (J.Cu.); chenjing765@163.com (J.Ch.)

**Keywords:** time-dependent damage, rock mass, *in situ* experiment, time-dependent damage model, damage rehabilitation

## Abstract

In underground tunnels or caverns, time-dependent deformation or failure of rock mass, such as extending cracks, gradual rock falls, *etc*., are a costly irritant and a major safety concern if the time-dependent damage of surrounding rock is serious. To understand the damage evolution of rock mass in underground engineering, an *in situ* experimental testing was carried out in a large belowground tunnel with a scale of 28.5 m in width, 21 m in height and 352 m in length. The time-dependent damage of rock mass was detected in succession by an ultrasonic wave test after excavation. The testing results showed that the time-dependent damage of rock mass could last a long time, *i.e.*, nearly 30 days. Regression analysis of damage factors defined by wave velocity, resulted in the time-dependent evolutional damage equation of rock mass, which corresponded with logarithmic format. A damage viscoelastic-plastic model was developed to describe the exposed time-dependent deterioration of rock mass by field test, such as convergence of time-dependent damage, deterioration of elastic modules and logarithmic format of damage factor. Furthermore, the remedial measures for damaged surrounding rock were discussed based on the measured results and the conception of damage compensation, which provides new clues for underground engineering design.

## 1. Introduction

Rock material evolves through a long-term geology process of hundreds of millions of years encompassing random voids and cracks of various scales. Therefore, the damage assessment of rock mass that has suffered engineering disturbance should be unique, as discussed by [1,2,3,4]. In underground construction, including metros, subsurface hydropower houses, highway tunnels and so on, the excavation of underground space changes the equilibrium of stress fields stored in rock mass and leads to rheological adjustment to a new stress equilibrium [5,6,7]. In the course of stress transition of rock mass and engineering construction, time-dependent failures of surrounding rock can become a costly nuisance and a major safety concern [8,9,10].

In recent years, the time-dependent damage properties of rock have been tested by indoor experiment using small specimens and *in situ* investigations in large tunnels or caverns [11,12,13,14,15,16]. Their achievements have exposed that the rock mass has obvious rheological characters. Although the rheological models for viscoelastic behavior and viscoplastic behavior of material have been developed successfully, little attention has been paid to rheological damage of rock, and even less concerning the damage correlation between time and strain. This is due to the limitation of rough field experiment conditions and imperfect experimental positions [14,17,18,19]. In practice, the time-dependent deterioration of material, such as reduction of elastic modules or decrease of strength, always occurs [20,21,22,23]. Thus, the time-dependent damage evolution of rock mass needs more in-depth efforts, especially *in situ* experimental tests.

The authors of this paper investigated the time-dependent damage of rock mass in a large scale tunnel in order to understand the damage evolution. This was detected in succession using ultrasonic detection after excavation and results indicated that the rock mass in underground tunnels exhibited time-dependent deterioration characteristics. Regression analysis revealed that the time-dependent evolution of damage factor followed the logarithmic function. To describe the exposed time-dependent damage characters of rock mass by *in situ* test, a damage viscoelastic-plastic model was presented. Furthermore, rehabilitation strategies of damaged rock, including the injection technique and pre-stressed rock bolt technique, were discussed to assist in withstanding the time-dependent deterioration of surrounding rock.

## 2. Information Regarding the Experimental Tunnel and Testing Method

### 2.1. Experimental Position

**Figure 1:**
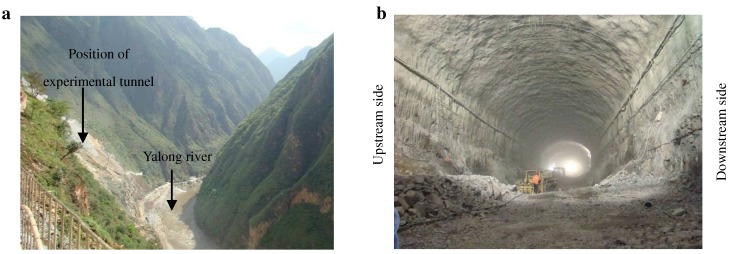
Jinping II experimental tunnel: (**a**) position of experimental tunnel; (**b**) general state of the experimental tunnel.

The experimental tunnel for damage testing was located at Jinping II hydropower station in Sichuan province, China. The size of the tunnel was 28.5 m in width, 21 m in height and 352 m in length at the time of experimental damage testing (Figure 1). The rock mass around the tunnel was Trias marble with three primary joint sets: (1) dip direction/amount of dip (20° ± 10°/79° ± 6°); (2) dip direction/amount of dip (70° ± 10°/80° ± 10°); (3) dip direction/amount of dip (50° ± 10°/30° ± 10°). The maximum initial geo-stress by hydraulic fracturing measurement in this tunnel was about 22.9 MPa.

**Figure 2:**
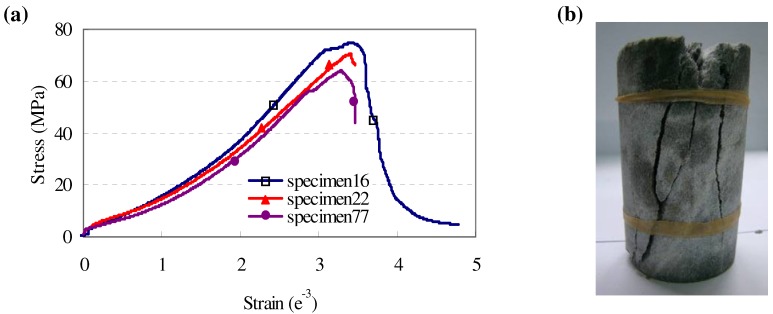
Laboratory experiment of intact marble specimen under uniaxial compression test: (**a**) strain-stress curves; (**b**) typical failure format and cracks of marble specimen.

A mechanical test of the marble in a laboratory showed that the uniaxial compression strength of intact marble specimen was about 70 MPa (Figure 2). The failure pattern of the marble specimen was split format. Damage testing of this rock was possible during the large scale excavation of this underground tunnel, considering the fact that the *in situ* strength of the rock mass was not more than half of the specimen’s strength and the concentrated stress of the surrounding rock after excavation was larger than 40 MPa. The basic mechanical parameters of the marble rock mass were 8–11 GPa in deformational modules and 0.23–0.26 in Passion’s ratio, as suggested by the project’s design institution.

### 2.2. Testing Instruments and Methods

The ultrasonic detection technique is often used to check rock damage, since the wave velocity is sensitive to internal micro cracks and voids of rock [24,25]. Here, we adopted the RS-ST01C ultrasonic instrument produced by RockSea Ltd. as the testing apparatus. The test of wave velocity was carried out using the method “one emission with two receipts”, *i.e*., using one emission-energy converter to send ultrasonic waves and two recipient-energy converters to receive waves at a distance of 20 cm [26]. Three converters were arranged along the hole’s axes, and their sequence was receipt converter, emission converter and receipt converter during the measuring process. The velocity of the longitudinal wave at the tested rock segment was calculated using Equation (1).

(1) v=LΔt

where *L* is the distance between emission energy converter and recipient energy converter; Δt is the traveling time of the longitudinal wave inside the rock mass.

**Figure 3:**
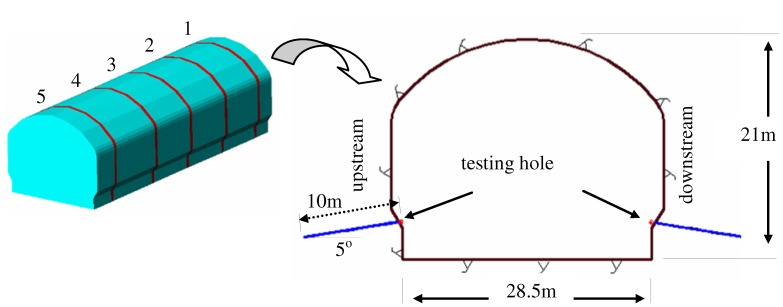
Testing sections and test holes for rock damage.

Five measurement sections were selected to measure rock damage along the axes of the tunnel with a distance of about 60 m. In these sections, the rock mass had similar joints density, efflorescent level, and rock classification. In each section, two testing holes were designed on the left side (upstream side) and right side (downstream side) of the tunnel respectively (Figure 3). The diameter of the test holes was 90 mm and their axes were vertical to the surface of the tunnel’s sidewall. Both of them were 10 m in depth and 5° in obliquity. As the converters moved in the test hole progressively along the axes in 20 cm steps, the data of rock velocity in different positions could be gained.

## 3. Analysis of Time-Dependent Damage

### 3.1. Basic Characters of Measured Ultrasonic Wave

**Figure 4:**
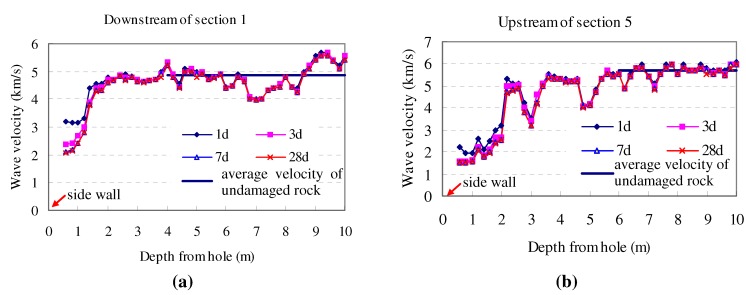
Measured ultrasonic velocity of rock mass in test holes.

After the tunnel had been excavated and the holes had been drilled, wave velocity measurements of the rock mass by RS-ST01C ultrasonic instruments began. The measurements were conducted on the 1st, 3rd, 7th, and 28th day. Thus, four measured curves of ultrasonic velocity with respective times were recorded for each test hole, as shown in Figure 4a,b. Considering the fact that there were many random joints in the rock mass, the measured velocity curves were not perfectly smooth. It is noted that the segment from 0 to 0.6 m could not be measured because the surrounding rock in this part was loose and cracked due to the surface of the tunnel being too close.

In Figure 4, the average wave velocity of undamaged rock mass was calculated by averaging the tested wave velocity in the end part of the test holes. The average wave velocity of intact rock was gained according to Equation (2) and tested data of wave velocity was chosen from the last 5 m of the test holes, which was the average wave velocity of the undamaged rock. Due to small differences in rock texture and various intensities of joints along the axes of the experimental tunnel, the average ultrasonic velocities were not similar.

(2) $$\bar{v^{o}}=\frac{1}{n}\sum_{i=1}^{n}v_{i}^{o}$$

where $\bar{v^{o}}$ is the average wave velocity of intact rock mass; $v_{i}^{o}$ is the measured wave velocity of intact rock mass in the test hole; n is the number of measured data.

All four curves in Figure 4 followed the same trend, *i.e*., each of them had a segment with low wave velocity at the beginning of the curves. Note that the lower velocity suggests a higher degree of rock damage; the *in situ* testing result reveals that there was less rock damage along the axes of the test hole from outside to inside.

Moreover, the ultrasonic velocity of wall rock decreased with time at the same position in the shallow segment, but there was not much difference in the deep segment; the joints, however, caused the roughness of the curve. This suggests that damage was time-dependent, especially for rock mass around the excavation space.

### 3.2. Time-Dependent Damage Evolution of Rock Mass

**Figure 5:**
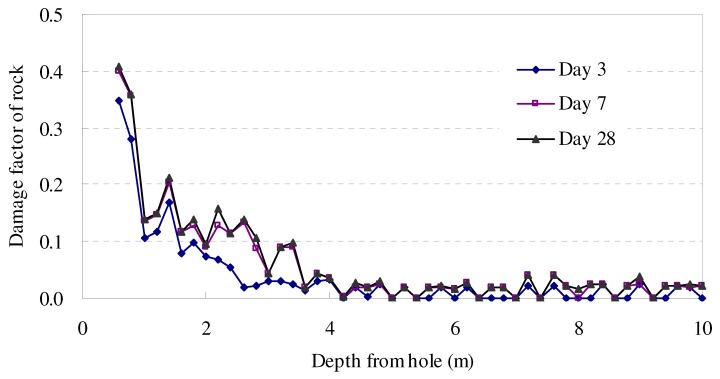
Time-dependent damage factor in Section 1 of experimental tunnel.

If we consider the measured data of wave velocity by ultrasonic detection at 1st day as the initial damage status of wall rock, the subsequent measured wave velocity of rock could be accepted as the time damage of wall rock. By comparing the subsequent three series of measured wave velocity results in the hole with the wave velocity at 1st day, the time damage factor ($D_{t}^{i}$) can be gained according to Equation (3) [27].

(3) $$D_{t}^{i}=1-{\left(v_{i}^{t}/v_{i}^{1}\right)}^{2}$$

where $v_{i}^{t}$ is the ultrasonic wave velocity of rock at i position on $t^{th}$ day; $v_{i}^{1}$ is the ultrasonic wave velocity of rock at i position on 1st day. This indicates that the time-dependent evolution of rock damage was apparent. For example, the time-dependent damage curve at the upstream testing hole of Section 1 is shown in Figure 5.

**Figure 6:**
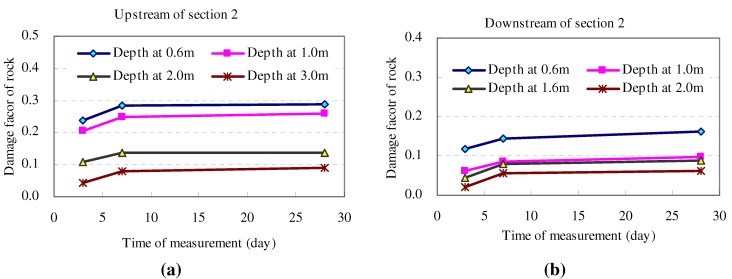

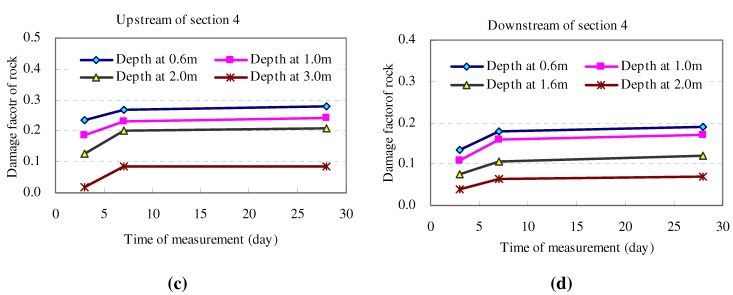
Time-dependent evolution of rock damage at various positions.

Analyzing the above damage factor curves, it is clear that wall rock damage changes degressively, similar to all measured results in other test holes. Figure 6 exposes the time-dependent damage process of rock mass at four selected depths, which followed a similar increase trend. This trend indicated that the time-dependent damage of rock can be described by some analytical functions. Kachanov (1958) and Broberg (1974) [27,28] suggested that functions of exponential, power and logarithm seemed to fit the time-dependent damage of material (Equation (4)), which had been studied and adopted also by Kowalewski (1994), Schulze (2001), Becker. (2002) [29,30,31]. However, the optimal function for describing the time-dependent evolution function for marble rock mass in Jinping II tunnel still needs to be verified.

Here, the time-dependent damage factor at the positions of 0.6 m and 2.0 m were adopted to show the process of regression analysis. Three functions in Equation (4), *i.e.*, Exponential, Power and Logarithm, were attempted to fit the data. The regressed results for functions are shown in Table 1.

(4) $$\{\;\begin{array}{l} \text{i})\;Logarithm:D_{t}=a\ln t+b\\ \text{ii})\;Power:\;D_{t}=at^{b}+c\\ \text{iii})\;\mathrm{Exponential}:D_{t}=ae^{bt}+c \end{array}$$

**Table 1:** Regression results of different functions.

Position (m)	Format	Residual
0.6	Logarithm	0.2155
	Power	0.4467
	Exponential	0.3148
2.0	Logarithm	0.0595
	Power	0.0695
	Exponential	0.0644

**Figure 7:**
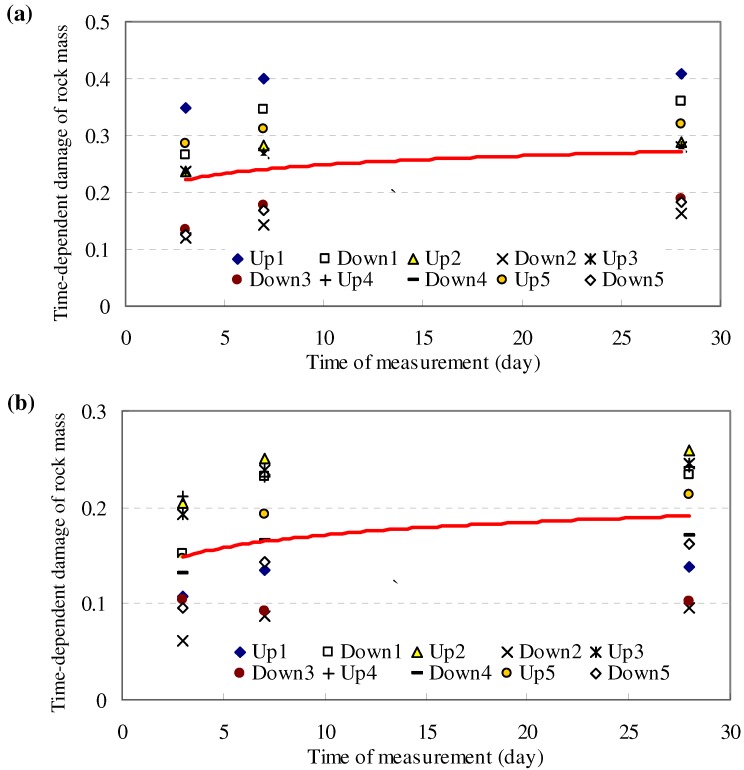
Fitting curve of time-dependent damage factor. (**a**) 0.6 m depth; (**b**) 2.0 m depth.

The residual value of different regressed functions indicated that the logarithmic function was the best expression of the three functions to describe the time-dependent evolution of rock damage in the Jinping II experimental tunnel (Equation (5) for 0.6 m position and Equation (6) for 2.0 m position). The regression analysis for damage equation had also been checked with the data of each tested hole (*i.e*., 10 holes). Most showed the same result, that the residual value of the logarithmic function was the smallest among these functions.The fitted result of measured damage factor showed that the logarithmic function can generally reflect the time-dependent process of rock damage (see Figure 7).

(5) $$D_{t,at\;0.6\;m}=0.0225\ln t+0.197$$

(6) $$D_{t,at\;0.6\;m}=0.0191\ln t+0.127$$

### 3.3. Time-Dependent Damage Mechanism

**Figure 8:**
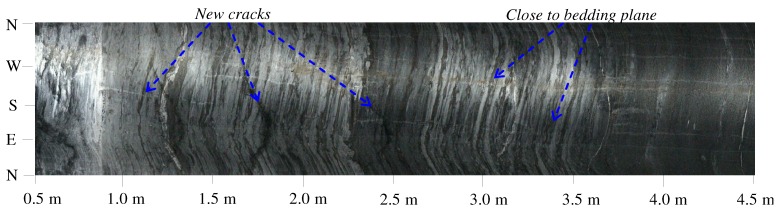
Cracks during creep damage observed by borehole televiewer.

Generally, rock mass is composed of intact rock and several joints or defects [32,33]. This means that the micro damage mechanism may be distinguished from the usual homogeneous materials. Observation using a borehole televiewer, a visual observing tool, was carried out in this experimental tunnel a month after excavation. Results showed that there were many cracks and lacunas with a width range from 1 to 3 mm in the testing hole. Furthermore, most segregated cracks were almost vertical to the axes of the test hole (as Figure 8).

The observed results indicate that the essence of time-dependent damage for rock mass was the development of micro cracks multiplying, extending and intersecting. Because the time-dependent deterioration of rock can be reflected by the wave velocity, which is related to elastic modules, as Equation (7) [34], the reduction of elastic modules can represent the time-dependent deterioration of rock mass.

(7) $$V_{p}=\sqrt{\frac{E(1-\nu )}{\rho (1+\nu )(1-2\nu )}}$$

where $V_{p}$ is velocity of longitudinal wave; E is elastic modules; ν is Poisson ratio; and ρ is density.

## 4. Numerical Descriptions

Above field testing of the underground tunnel exposed several time-dependent characteristics of rock mass, which included: (1) Surrounding rock displayed time-dependent damage, which tended to tamper out; (2) Ultrasonic wave testing and televiewer observation indicated that the essence of the rock’s time-dependent damage was the reduction of elastic modules induced by crack development; (3) The damage factor relating to time corresponded with logarithmic functions.

**Figure 9:**
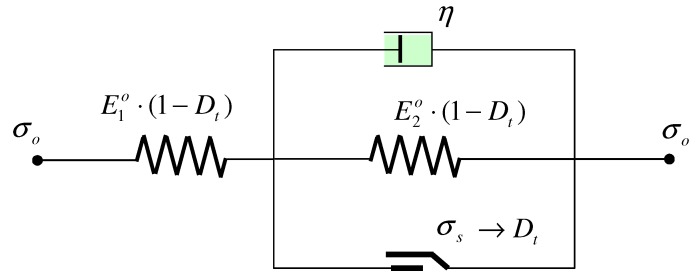
Cell structure of the damage viscoelastic-plastic model.

To describe the above damage characters of the surrounding rock in the underground tunnel, a damage viscoelastic-plastic model (DVEP) was proposed to modify the acknowledged viscoelastic model and integrate a time-dependent damage factor. The structure of the DVEP was such that a Kelvin element was connected by a plastic element in parallel format, and a spring element was connected to this in series format, as shown in Figure 9. In the DVEP, each spring added a time-dependent factor ($D_{t}$). Bearing in mind that the time-dependent damage factor is generally related to strain [14,35,36], the $D_{t}$ was assumed to be as in Equation (8) after absorbing the format of Equation (5). In Equation (8), both the time-dependent and strain-dependent damage were tentatively considered.

(8) $$D_{t}=a\ln(t)+b\varepsilon^{p}$$

If the effective stress ($\sigma_{o}$) is lower than the yield stress ($\sigma_{s}$), time-dependent damage factor $D_{t}$ equals “0” and the constitutive equation of this model can be expressed as Equation (9).

(9) $$\sigma +\frac{\eta }{E_{1}^{o}+E_{2}^{o}}\dot{\sigma }=\frac{E_{1}^{o}E_{2}^{o}}{E_{1}^{o}+E_{2}^{o}}\varepsilon +\frac{E_{2}^{0}\eta }{E_{1}^{0}+E_{2}^{o}}\dot{\varepsilon }$$

If the effective stress ($\sigma_{o}$) is not lower than the yield stress ($\sigma_{s}$), a time-dependent damage factor needs be added and the constitutive equation can be expressed as follows.

(10) $$\sigma +\frac{\eta }{(1-D_{t})E_{1}^{o}+E_{2}^{o}}\dot{\sigma }=(1-D_{t})\frac{E_{1}^{o}E_{2}^{o}}{E_{1}^{o}+E_{2}^{o}}\varepsilon +\frac{E_{2}^{o}\eta }{E_{1}^{o}+E_{2}^{o}}\dot{\varepsilon }$$

By using the Laplace conversion on the premise that the $D_{t}$ is constant in each iterative step during difference calculations simplifying the change process, the damage creep equation can be gained, as

(11) $$\varepsilon =[\frac{E_{1}^{o}+E_{2}^{o}}{(1-D_{t})\cdot E_{1}^{o}{E_{2}^{o}}_{1}}-\frac{1}{E_{2}^{o}(1-D_{t})}\exp(-\frac{E_{2}^{o}(1-D_{t})}{\eta }t)]\cdot \sigma_{o}$$

Equation (11) shows that this model can describe the transient elastic deformation and steady viscoelastic deformation if the effective stress ($\sigma_{o}$) is smaller than the yield stress ($\sigma_{s}$). Equation (11) also shows that this model can describe damage transient elastic deformation and damage viscoelastic deformation if the effective stress ($\sigma_{o}$) is larger than the yield stress. Thus, the DVEP can express the characters of surrounding rock exposed in the test tunnel, *i.e*., convergence of creep damage, deterioration of elastic modules and logarithmic format of damage factor.

By translating the one dimensional constitutive equation to three constitutive equations, a numerical code can be developed to simulate the time-dependent damage of underground tunnel [37]. During the translation of constitutive equations, the $D_{t}$ is assumed as constant in each iterative step for simplifying the calculation process in the small differential time, and special substitutes should be considered from elastic modules (*E*) to bulk modules (*K*) and shear modules (*G*). The final 3D constitutive equation is expressed in Equation (12).

(12) $$\varepsilon_{ij}(t)=(\frac{1}{2G_{1}^{o}}S_{ij}+\frac{1}{3K_{1}^{o}}\sigma_{m}\delta_{ij})\frac{1}{1-D_{t}}+\frac{1}{2G_{2}^{o}(1-D_{t})}[1-\exp(-\frac{G_{2}^{o}(1-D_{t})}{\eta })t]S_{ij}$$

**Figure 10:**
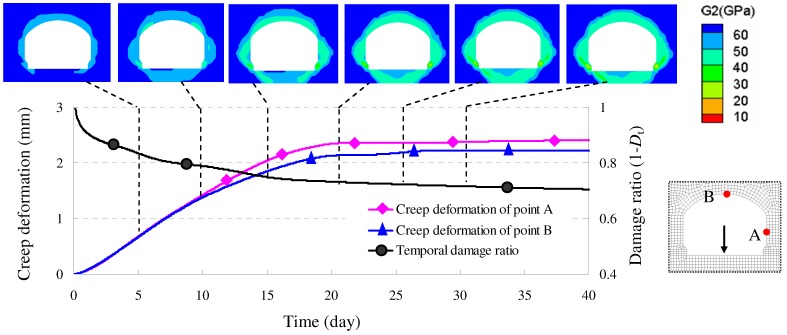
Simulated time-dependent damage of test tunnel by damage viscoelastic-plastic model (DVEP).

Simulation of above testing tunnel shows the developed model, *i.e*., DVEP, can distinctly express the time-dependent damage evolution process of surrounding rock, as shown in Figure 10.

**Figure 11:**
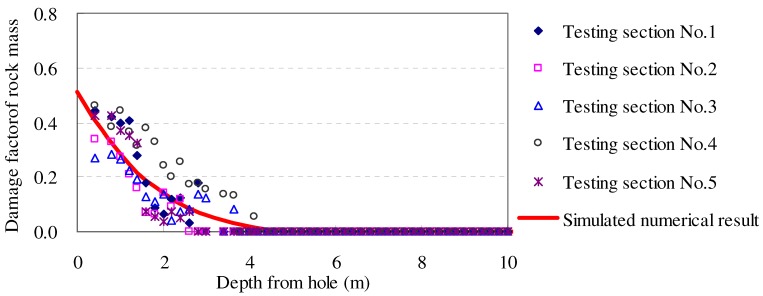
Comparison of the simulated numerical damage factor and the tested damage factor of rock mass along the borehole after 30 days.

The simulated result indicated that the DVEP can reflect the damage of rock mass not only in spatial distribution but also in time-dependent development, which is in accordance with the field testing results (see Figure 11).

## 5. Damage Rehabilitation of Rock Mass

Since time-dependent damage of surrounding rock reflects the actual behavior in underground engineering, it is necessary to rehabilitate this deterioration of wall rock for the purpose of maintaining the long-term safety of the tunnel after opening. Considering that the mechanical changes of micro cracks and micro holes were the reason for the rock damage, reducing or repairing micro cracks and micro holes is necessary. As a reference, we adopted the geometry definition of material damage as suggested by Kachanov [27].

(13) D=1−A¯A0

where $A_{0}$ is the initial cross area of non-damaged material; $\bar{A}$ is the effectual loading area of damaged material. If we accept the viewpoint that the indirect definition of damage factor by wave velocity is equal to the geometry definition of damage factor by effectual loading area, the wave velocity of rock mass was directly proportional to the effectual loading area in damaged rock (as in Equation (14)).

(14) $$v_{i}\propto \bar{A}$$

Thus, the rehabilitation of wall rock can be achieved by increasing the effective loading area of rock mass. As a representative friction-type material, strength of rock mass satisfied the Mohr-Coulomb criterion [38].

(15) $$\tau =C_{0}+\sigma_{n}\tan\phi$$

Where, $C_{0}$ is the cohesive strength of material; $\sigma_{n}$ is the normal stress at friction plane, ϕ is the friction angle of material. To improve the effectual loading strength of damaged rock mass, two techniques are feasible:

(1) Injection of liquid cement in damaged rock can be used to amend $C_{0}$ and ϕ of wall rock, which can solidify the cracks and fill the micro holes;

(2) Pre-stressed rock bolt technique can be used to minimize the normal stress on cracks and restrain further splitting or expanding of cracks.

**Figure 12:**
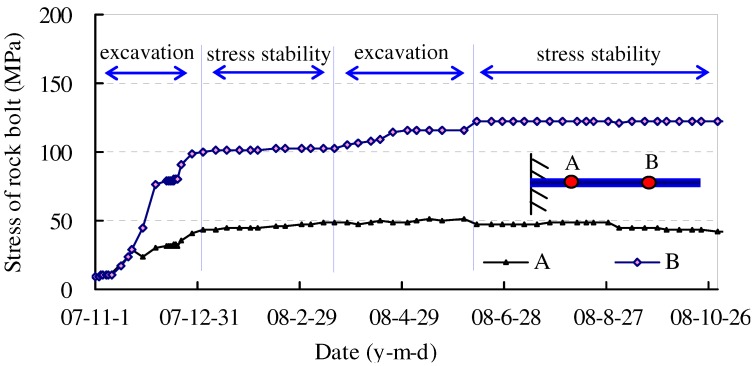
Stress curve of inspected rock bolt under stable state of surrounding rock.

The function of the above engineering techniques in improving the effectual loading area for wall rock can be tested by ultrasonic detection according to Equation (7). In practice, the effect of these rehabilitation techniques can be reflected indirectly by inspecting rock bolt stress. If this remains stable (see Figure 12), the mechanical state of wall rock is constant. Otherwise, new damage of wall rock would induce further mechanical deterioration and increase bolt stress with time.

## 6. Conclusions

In underground engineering, time-dependent damage of rock mass is common causing either accelerated damage or slowdown damage. In this *in situ* experimental investigation of rock damage using ultrasonic wave tests, no accelerative time-dependent damage had been observed, only slowdown time-dependent damage had been observed. Furthermore, the measured results of wave velocity at different times showed the damage factor of surrounding rock was time-dependent. The regression of testing data also indicates that the damage evolution of rock mass generally followed the logarithmic function.

To describe the exposed time-dependent deterioration of rock mass by field test, a new damage viscoelastic-plastic model was presented, which can embody these observed behaviors of rock, such as convergence of time-dependent damage, deterioration of elastic modules and the logarithmic format of damage factor.

Given that rock mass is a typical friction material, the rehabilitation of damaged rock can be achieved by amending the physical properties and increasing the normal stress according to the required damage reparation. Injection of liquid cement in damaged rock and installation of pre-stressed rock bolt on rock surface are two practical ways to achieve this.
